# Ethical, legal and societal considerations on Zika virus epidemics complications in scaling-up prevention and control strategies

**DOI:** 10.1186/s13010-017-0046-8

**Published:** 2017-08-25

**Authors:** Ernest Tambo, Ghislaine Madjou, Christopher Khayeka-Wandabwa, Oluwasogo A. Olalubi, Chryseis F. Chengho, Emad I.M. Khater

**Affiliations:** 1 Department Biochemistry and Pharmaceutical Sciences, Higher Institute of Health Sciences, Université des Montagnes, Bangangté, Cameroon; 2 Africa Disease Intelligence and Surveillance, Communication and Response (Africa DISCoR) Institute, Yaoundé, Cameroon; 3 Schoolof Pharmaceutical Science and Technology (SPST), Health Sciences Platform, Tianjin University, 92 Weijin road, Nankai District, Tianjin, 300072 People’s Republic of China; 4 Africa Population and Health Research Center (APHRC), Nairobi, Kenya; 5 Department of Public Health, Kwara State University (KWASU), Malete, Nigeria; 6 Department of life Sciences, Coventry University, Leicester, UK; 7 Public Health Pests Laboratory (PHPL) of Jeddah Governorate, Jeddah, Saudi Arabia; 8 Department of Entomology, Faculty of Science, Ain Shams University, Cairo, Egypt

**Keywords:** Zika virus, Epidemics, Ethical, Legal, Abortion, Mental, Reproductive, Programs

## Abstract

Much of the fear and uncertainty around Zika epidemics stem from potential association between Zika virus (ZIKV) complications on infected pregnant women and risk of their babies being born with microcephaly and other neurological abnormalities. However, much remains unknown about its mode of transmission, diagnosis and long-term pathogenesis. Worries of these unknowns necessitate the need for effective and efficient psychosocial programs and medical-legal strategies to alleviate and mitigate ZIKV related burdens. In this light, local and global efforts in maintaining fundamental health principles of moral, medical and legal decision-making policies, and interventions to preserve and promote individual and collectiveHuman Rights, autonomy, protection of the most vulnerable, equity, dignity, integrity and beneficence that should not be confused and relegated by compassionate humanitarian assistance and support. This paper explores the potential medical and ethical-legal implications of ZIKV epidemics emergency response packages and strategies alongside optimizing reproductive and mental health policies, programs and best practice measures. Further long-term cross-borders operational research is required in elucidating Zika-related population-based epidemiology, ethical-medical and societal implications in guiding evidence-based local and global ZIKV maternal-child health complications related approaches and interventions. Core programs and interventions including future Zika safe and effective vaccines for global Zika immunization program in most vulnerable and affected countries and worldwide should be prioritized.

## Background

Zika virus is a mosquito-borne infection mainly transmitted via the bite of *Aedes* mosquitoes (*Ae. Aegypti* and *Ae.Albopictus*), the same species that transmit dengue, yellow fever, West Nile and Chikungunya). These mosquitoes species has already spread to 31 countries and territories in Latin America and the Caribbean. Until 2015, Zika had rarely appeared in the Western hemisphere. However, the recent rapid spread was declared a public health emergency of international concern by the World Health Organization (WHO) on February 1, 2016, due to reported outbreaks and ongoing transmission incremental trend with public phobia of hinted potential linkage to cases of congenital microcephaly, neurological disordersand still-birth in Brazil, Columbia and other ZIKV-affected countries including sub-Saharan Africa, Middle East and parts of Asia-pacific areas [1–4]. It is perhaps difficult, if not impossible to screen all human reservoirs with the prevailing asymptomatic and subsyndromic co-infections. Nonetheless, more evidence to decipher the molecular basis of the virus, research endeavor to include pregnant women case-control studies to compare rates of Zika infection in babies who are born with increasing risk of microcephaly and in those without it, genetic sequencing of the virus and efforts to develop a molecular diagnostic test for Zika infections are ongoing in affected populations [5, 6].

Much of the fear and uncertainty around Zika stems from a potential linkage between pregnant women infected with ZIKV and risk of their babies being born with microcephaly, a birth defect characterized by an abnormally small head and brain damage [7–9]. Nevertheless, more research is needed to elucidate the role of reservoir-hosts seroconversion in transmission dynamics, influence of innate versus acquired arboviral immunity, early diagnosis and long-term physio-pathological consequences of ZIKV infections on maternal and fetal-childhood development. The intriguing aspect of the disease lies on the fact that Zika virus often causes minimal (or no) symptoms in infected patients. Pregnant mothers might not need to have symptoms to transmit the virus to their fetuses, and there is neither reliable nor prompt point of care diagnostic test nor treatment and vaccine to combating arboviral diseases. Equally, there is limited steady and proven advice on local/international travel or tourism; especially to places where the virus is circulating or transparent and equitable actions in morally acceptable alternatives with limited or without harms “*do no harm*” versus “*precautionary principle or uncertainty in justification*” to the family/population as a whole benefits [10].

The Centers for Disease Control and Prevention (CDC) issued a travel alert for pregnant women, advising them to postpone travel to areas where ZIKV transmission is ongoing [11]. The CDC has since created an algorithm for testing pregnant women who may have been exposed to the virus, but it is exceedingly complicated and more troublingly for it may lead to the detection of microcephaly outside the window period of birth defect when it is too late to do anything about it resulting to possible pregnancy termination [10, 12, 13]. In the prevailing scenario, when it does happen that there is detection of ZIKV infection and risk of potential birth defect to a pregnant mother exposed to ZIKV, what is next? What is the appropriate medical decision or advice to the pregnant mother or couple? Does the currently available evidence supports the hypothesis that prenatal ZIKV infection is a cause of microcephaly and other brain anomalies? Are we taking the right action or are we applying the most effective solutions? So far, two lines of seemingly compelling evidence support a link between the virus and microcephaly. First, in an epidemiological study conducted during the outbreak in Brazil, 88 pregnant women who had an onset of rash in the previous fivedays were tested for ZIKV genetic material. Among the 72 women who had positive tests, 42 underwent prenatal ultrasonography, and fetal abnormalities were observed in 12 (29%); none of the 16 women with negative tests had fetal abnormalities [6, 9]. The abnormalities that were observed on ultrasonography varied widely, and some findings lacked postnatal confirmation because the pregnancies were on course. Secondly, in a complementing retrospective analysis after the 2013–2014 outbreak of Zika disease in French Polynesia identified eight cases of microcephaly; the authors used serologic and statistical data and mathematical modeling to estimate that 1% of the fetuses and neonates who were born to mothers who had been infected with ZIKVin the first trimester had microcephaly [6, 14], a prevalence that was approximately 50 times as high as the estimated baseline prevalence. However, this estimate was based on small numbers, confidence intervals were wide, and the risk of other adverse outcomes (e.g., other brain anomalies) was not assessed [6].

With different laws and constitutions in Latin America, The America, Africa and other Zika-affected countries with increasing potential ethical, medical  violations and legal issues, it is difficult to approve local context legal policies, upholding Human Rights under the UN Conventions on women and child health, Rights of the Child and Disabilities framework perspectives. But also, governments and professionals shared responsibilities to abide by the International Health Regulation 2005, ethical deontology/ consequentialism and standard practices to protect women experiencing reproductive age pregnancies delay for the foreseeable future (or abortion), fostering proper and robust case-control evidence and discussions on association between ZIKV infection, Human Rights, maternal and child health promotion [6, 15]. Furthermore, despite accumulating evidence that supports the link between ZIKV infection and microcephaly, most experts have taken care not to state that ZIKV infection is causally related to these adverse health outcomes [15, 16] leaving majority of the vulnerable at cross roads and suspicion.

**Fig. 1:**
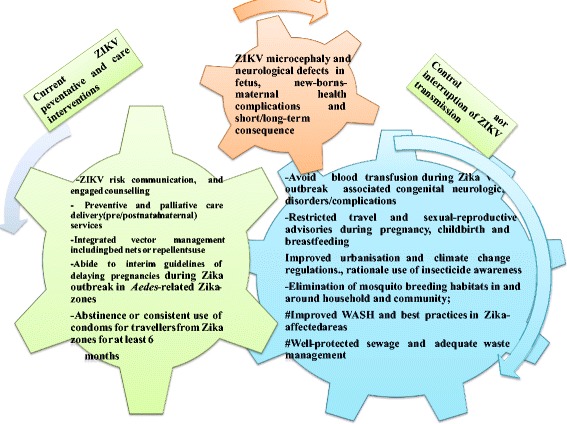
Contextual Zika virus (ZIKV) interlocking complications prevention and control strategies and interventions

This paper addresses ethical, legal, medical and societal issues alongside implications of ZIKVepidemics risk and devastating consequences on women and fetal growth and development. It further advocates innovative solutions for strengthening the sexual and reproductive health, and mental health preventive and control measures alongside travel and transfusion medicine programs and interventions strategies. We highlight key urgent contextual issues surrounding ZIKV interlocking complications, and scaling-up prevention and control strategies (Fig. 1).

### Improving *Aedes*-Mosquito vectors transmission interruption and better access to quality health care services

Determined, sustainable and time frame structured multi-facet actions should urgently be taken in the widely affected areas in strengthening the role of respective governments towards enhancing ZIKV preparedness of local health systems [6]. Complementary strategies are recommended relating to open access to Zika information and knowledge, vulnerable population *Aedes* mosquito and ZIKV complications awareness and resilience outreach mainly pregnant women. Effective uptake and utilization of preventive measures including mosquito protection and contraceptive methods, counseling services and information dissemination, reproductive and social education and awareness strategies reinforcement in modern era of integrated  vector control and care management in the context of microcephaly and a range of congenital neurological abnormalities associated with Zika epidemic impact are also highly desired. These efforts can be tapped through reinforcing mandates of rapid response initiatives from the WHO and other agencies. Moreover, existing ethical, medical, cultural and societal issues in some affected settings might be due to public traditional and cultural practices, beliefs or myths, misconception and fear linked to resistance, intense urbanization and migration, crowding, poor sanitation and hygiene those should be addressed [4, 7, 17, 18]. To date, the best preventive measures against ZIKV include but not limited to elimination of *Aedes* mosquito larval water habitats in and around household and buildings under contruction including removal of air-conditioning drainage buckets, barrels and abandoned car tyres, improved hygiene and regular sanitation, good water storage containersand other backyard containers or ornamentals, well-protected sewage and adequate waste management that can provide potential mosquito breeding sites [18].

### Strengthening Zika and pregnancy related medical care capacity and interventions

The mainstays of ZIKV infection management are immediate clinical and laboratory examinations followed by appropriate medical and psychosocial counselling, bed rest and supportive care [19]. ZIKV and other arbovirus species co-circulation and expansion resulting on specific viral seroconversion and seroprevalence is still poorly understood and require sentinel/population-based surveillance and asymptomatic reservoir diagnostic screening. If available, this can be important in anticipating, preventive measures and timely acute asymptomatic or syndromic complications management [15]. The global community has the responsibility to protect and promote the health of all people as stated in the first international Alma Ata declaration underlining the importance of primary healthcare and Human Rights [20]. The risk of microcephaly and neurological defects for many unborn children is far too great to justify any avoidable trip in *Aedes*-linked ZIKV settings or seek advice from medical providers in line with follow the WHO and CDC recommendations and guidelines. Most affected governments and public health authorities should provide reliable, consistent and trustful evidence-based information and communication in rebuilding  population trust, care-seeking or practicing positive behavior and attitude changes [4, 9, 21, 22]. This also include advice to returning travelers from Zika-affected areas to practice correct and consistent use of condoms and safer sex or at least 6 months sex abstinence with their partner [22]. Authors emphasizes on the significance of parents and tourists awareness and advice, pre-natal and antenatal Zika information related sexual and reproductive health education outreach mainly during early stage of pregnancy as part of public health messaging of critical importance. Also, it is still ofconcernto think of the unknown future implications of Zika-related pathophysiological, emotional and societal consequences such as trauma, stress, depression, stigmatization and social withdrawal impacts on the affected populations and nationwide socio-demographic and economic over time yet consortia of grey areas are unaddressed where:

i) Epidemiological studies are often complex because Zika causes a relatively mild illness in adults and there is no widely deployed test for the virus. This means that most of the mothers who have participated in previous studies were never diagnosed with Zika, even if they contacted it. To address this problem-there is a critical need for development and deployment of more sensitive field adaptable and point of care Zika diagnostics to national/regional clinical reference laboratories.
ii) As a responsiverather than reactive measure and would require that ethically and socially sound obstetricians and medical psychologists should advice couples and patients on very difficult decisions involving risk to ongoing or planned pregnancies throughout *Aedes* associated Zika-affected and prone settings [15, 16].
iii) Proponents of delayed pregnancy [23] ought to realize and appreciate from both economic and political perspective, altered birth cohort progression throughout the region, coupled with disabled care, may have long-term disruptive political, systemic and economic impacts in these countries.

### Ethical issues related to ZIKVepidemics on family planning, sexual-reproductive policies and intervention measures

To date, there is no vaccine or treatment available for the ZIKV and no known treatment for children who suffer brain damage in the womb.The Caribbean and Asia-Pacific-ZIKV-prone regions aiming at preventing vertical mother-to-child transmission impacts require innovativestrategies including expanded protective sexual and reproductive health measures against their aspirations for freedom and liberty in most affected populations [6, 24]. With concrete association between Zika infection and birth defects, leading concerned health authorities around the globe like the Pan American Health Organization (PAHO), WHO and CDC had issued guidance to pregnant women and those seeking to become pregnant toconsider delaying travel to Zika-affected areas, and for those living in countries with wide-spread Zika transmission to avoid mosquito bite exposure and/or having unprotected sexual intercourse with suspected traveler/tourist, abortion, or maintenance of confidentiality in risk situations from *Aedes*-related Zika-prone settings [6, 25]. In some countries public health authorities havegone even further, recommending that women should postpone becoming pregnant for a period of time; most notably, the Minister of Health of El-Salvador, a country which is experiencing a rise in suspected Zika cases, has recommended delaying pregnancy until 2018. In addition, there is an urgent need to scale up integrated vector control programs including appropriate and effective preventative measures to reduce and interrupt mosquito bites exposure and sexual or blood transfusion transmission of ZIKV.

In such rapid circumstantial opinions, decisions and directive are prone to bring to perspective socio-cultural challenges on a number of grounds: a) Many women across the affected regions havelimited access to contraceptives and other reproductive health services, b) Most of the womenexperiencehigh rates of sexual violence as much as they do face otherreproductive health decision-making barriersthat can result in unintended pregnancies and c) Some of these Zika-affected countries have among the strictest abortion laws in the world [26–28]. All these put in perspective potentially present women at risk with a challenging ethical, legal and psychosocial situation [27–29].

Instead of focusing on unplanned directives with no strategy towards addressing context of psychosocial implications, it would be more valuable to reinforce family planning and other sexual-reproductive health programsintegration coupled with innovative integrated vector surveillance and management strategies. Implementing contextual and evidence-based *Aedes* vector breeding sites reductions and biting interruption interventions should have positive outcomes on the most vulnerable groups of maternal and child health (MCH). Strengthening existing local sexual and reproductive health education and awareness outreach resilience  and preventive measures as well as improved blood transfusion screening programs in remote health community centers are needed in as much as addressing ZIKV knowledge gaps and issues:

i) We are neither able to forecast the next epidemic nor efficiently prepare for its effects onthe population. What would be tenable is to ensure that adequate reproductive health policies are put in place removing all barriers preventing access to holistic services to women of all sectors of society in a timely manner.
ii) In the light of these developments, we believe that prompt actions to resolve ethical-legal questions and issues are warranted amongst vulnerable local community, governments and stakeholders. Investing on *Aedes* associated ZIKV advanced operational research is urgently needed to realign evidence-based local and global policies, legislation and rights in tune with the internationally treaties and standards as well as affected community’s unmet needs in Zika complications context. For instance, the UN’s third sustainable goal-target 3.7 aims to ensure healthy lives and promote wellbeing-reads: “By 2030, ensure universal access to sexual and reproductive healthcare services, including family planning, information and education, and the integration of reproductive health into national strategies and programmes” [30] yet reproductive rights in a number of countries and regions have remained deterrent [26].
iii) Supporting organizations acting on population growth such as Population Matters (www.populationmatters.org), Population and Sustainability Network (www.populationandsustainability.org), and PopOffsets (www.popoffsets.org), which help people and organizations to offset their environmental sustainability needs by funding family planning around the world. Through this we will be able to live the spirit of integrated vector management and control from environmental, population awareness, medical and reproductive health performance and cost-effectiveness.
iv) Fostering Aedes linked ZIKV modeling-based approaches and applications can support effective health programming and providing further understanding of spatio-temporal risk and drivers interactions, transmission dynamics and spread pattern for evidence *Aedes* related ZIKV and other arboviral diseases prevention and control.

### Addressing ZIKV epidemics complications and abortion issues

Zika outbreakscontinue to raise concern and fear on worsening maternal and child health in most developing countries [3, 4, 9]. However, little is understood of human-vector-environment risk factors/determinants and interface on specific epidemics emergence and spread  impact on prognosis, effective prevention and control are still poorly elucidated and is imperative in predictive and preventive strategies and measures. Particularly, fears of rise in deaths from unsafe abortions as campaigners urge governments in Latin America to rethink bans on abortion and make contraception widely accessible and available. Campaigners are calling on Latin American governments to rethink their policies on contraception and abortionthat could lead to serious complications and varied degrees of disability later in life. This is because it is argued that ZIKV spread by sexual intercourse and potentially via shared injections and 'infected patient saliva'; scenarios that might lead to intensification of ZIKV transmission course and rise in women’s infection incidences leading to risky, unsafe and illegal abortion related consequences [3, 4, 31, 32]. Further, exploration and investigation of population-based behavioural andecological risk factors or determinants and impacts on the changing and rising emerging infectious disease epidemiologic changes burden at different levels and settings is critical in early warning risk indicators and better innovative health programming to *Aedes* related Dengue and ZIKV planning, mitigation and prevention programs and strategies. A consortium of Experts used the Bradford Hill’s causality framework assessment that provided ZIKV associated congenital microcephaly and neurological complications consensus reports as a basis for global health response. The basis documented that with the pregnant women affected and foetal abnormalities mostly in the first trimester by the circulating ZIKV infection crossing the placenta and replication in human brain were associated with risk of stillbirth and congenital defects in the Pacific region, The Americas and West Africa [5, 6, 25–28]. Likewise, Zika affected countries in the Pacific and the Americas reported temporal association of ZIKV infection preceding the onset of Guillain-Barré Syndrome or GBS similar to ZIKV outbreak in French Polynesia in 2013–2014, where one in 4000 ZIKV patient developed GBS [17, 18, 26–28]. More ZIKV transmission and complications awareness campaign to communities on the risk of Zika, acute and severe consequences alongside effective and reliable psychosocial measures, neuroimaging monitoring and medical-legal approaches in early stage antenatal care is also observed to be crucial in public health preventative measures [4, 6, 27–30]. Hence, there is a need that every infant born from pregnancies with possible recent exposure to Zika or primarily *Aedes* mosquito-borne virus association with brain abnormalities should receive appropriate postnatal imaging and testing for Zika to ensure proper and timely care. Prior adversities have a wealth of insights to share [33] for example, the rubella epidemic congenital syndrome during pregnancy, occurred between 1964 and 1965 in the United States and led to 12.5 million cases. A total of 20,000 children were born with congenital syndrome (11,000 deaf, 3500 blind and 1800 mentally retarded, and 2100 neonatal deaths and more than 11,000 abortions) [33].

The presented insights call for more proactive and cost-effectivepublic health and travel medicine programs and strategies that highlight the need for continuous and coordinated ZIKV research and related epidemic contexts elucidation for evidence-based expansion of integrated vector control programs, reinforcement access to contraception and blood donor screening against arboviral diseases and microbial resistanceprogressive solutions. Moreover, there is need to scale-up rural and remote community health professional care services delivery and laboratoryfacilitiesparticularly for low income and marginalizedgroups [3, 5, 7, 34, 35]. Also, to extend intensive awareness campaign to communities on the Aedes risk factors, acute and severe ZIKV infection consequences/complications alongside suitable and effective countermeasures should readily be available supported by health and legal counseling services in ZIKV-affected countries similar to effective and reliable psychosocial measures, and medical-legal response strategies against Rubella epidemics [7, 9, 33, 36–38].

### Poverty-related ZIKV epidemics consequences on mental health and transfusion medicine

ZIKV primary route of transmission is bythe *Aedes* mosquito bites, although the extend from other routes of transmission require further research such as human-mosquito-human and human-to-human via sexual transmission and bodily fluids transmission (e.g., semen, saliva, urine, breast milk) [39–41]. With very scarce access to mental health professionals, shortages in most affected countries can lead to more stigmatization, panic and anxiety stress, depression, to suicidal and other social issues in pregnant women and affected communities. Addressing epidemiologic, clinical knowledge and skills gaps and ZIKV complications issues requires urgent sustained funding support on new and appropriate evidence-based long-term contextual programs and mitigation measures, survivor (s) and family educational and psychosocial counselling to psychiatry rehabilitation activities. An early spectrum of less severe brain damage among infants have correlated with ZIKV, but these conditions can only be detected well after birth when cognitive testing can be conducted over time [8, 20, 41, 42].

Public health challenges and problems have been left unaddressed for the developing countries and lack of running water and waste management in conjunction with urban crowding and poor housing. These factors coupled together have given rise to the perfect set of conditions for the transmission of such mosquito-borne viruses [43–45]. Still, the Brazilian authorities recommended preventive and protective measures against ZIKV infections during the February 2016 Rio Carnival attended by more than 500,000 visitors, and additional evidence-informed guidelines were drawn to protect all vulnerable local populations and frontline humanitarian workers, visitors in affected countries and players at the Olympic and Paralympics games in September 2016, in Brazil [46, 47]. In addition, sexual and reproductive transmission of the virus from male-female partners, homosexuals or sex workers for undefined periods of time after the partner’s infection as well as maternal-child health ethical, medical and societal knowledge gaps and implications require further research in rolling out future Zika vaccination programs [7, 44, 47–51].

## Conclusions

Effective and coordinated response to ZIKV infection and its serious complications is urgently needed to gain a comprehensiveelucidation of public health Zika-related issues for evidence-based improvements on local and global health laws and regulations on the rationale use of pesticide (e.g. aerial insecticide application). Moreover, proactive efforts should be devoted to reducing *Aedes* linked Zika vulnerability and infections made possible through aggressive public/community awareness, readiness to optimizing integrated vector management (IVM) programs, early ZIKV diagnosis, and counselling and control policies and intervention best practices. Moving forward will require engaging the global community including all stakeholders to advocate and participate in strengthening local and national and global *Aedes* and ZIKV and other arboviruses surveillance data sharing and public access, defining early-warning indicators for risk communication in public/community engagement, vigilance and resilience. Leveraging on digital technology and service to scale up timely and effective *Aedes* and ZIKV epidemiologic, entomological and laboratory surveillance, timely *Aedes* mapping and ZIKV data sharing, and risk communication are imperative in guiding evidence-based ethical, legal and societal practical and acceptable recommendations and guidelines; but also in protecting the life and Rights of most vulnerable populations and communities worldwide. Likewise, sustainable and proportionate operational research and development on innovative approaches and interventions especially safe and efficacious Zika vaccines are urgently needed to support local, regional and global mosquito IVM programs. Just as health workers, medical practitioners and public health experts are working to address key determinants regarding testing, screening, treatment, vaccination and prevention of ZIKV. Equally, lawyers, ethicists, legislators and policy-makers are assessing emerging legal and ethical issues. Legal triage entails prioritization of these issues in real-time to facilitate legitimate rights public health and medical responses by: (1) identifying enabling and disabling issues; (2) gauging changing legal and ethical norms; (3) crafting and explaining innovative solutions; and (4) consistently revisiting the utility and efficacy of legal guidance. Profound reproductive rights issues surround access and use of contraception and abortion, especially among pregnant minors. Many of the nation’s experiencing widespread ZIKV infections have religious foundations and related laws that deeply conflict with these services. Strengthening ZIKV screening recommendations may apply to at-risk groups like infants born to mothers or non-immune traveler with exposure history and or contact (sexual or uncheck blood transfusion) from infected patient from endemic regions. Potential for discrimination arises as mothers (e.g., migrants from Mexico) are targeted for screening. The 2016 Summer Olympics and Paralympics games in Brazil, Muslims Hajj and Umrah mass-gathering pilgrimage in Saudi Arabia and continued mosquito spread to North America heighten the potential for rapid global extents of ZIKV and other arboviral infections. WHO has expressed disdain for any national travel bans or restrictions? The CDC has issued recommendations and voluntary travel warnings for pregnant women and vulnerable population in affected countries as well as tourists. As seen in response to EVD and SARS, other nations may impose restrictive travel policies on their citizens or attempt to keep out or screen persons arriving from “hot spots ZIKV zones”. Furthermore, investing on ZIKV R&D for more sensitive and field or point of care diagnostic for operational population-based screening, especially men and women at reproductive age, fetus and younger children for ZIKV at birth and during the course of development monitoring data sharing programs  in *Aedes*-prone and affected settings. Conversely, if efficacious treatments for ZIKV infections among infants become a part of the standard of care practices, failures to screen at risk infants may lead to liability later if their disabilities could have been prevented to future Zika safe and effective vaccines for global Zika immunization program.

## Abbreviations

CDC: Centre for disease prevention and control
EVD: Ebola virus disease
PAHO: Pan american health organization
SARS: Syndrome acquired respiratory disease
UN: The United Nations
WHO: World Health Organization
ZKIV: Zika virus
